# A Standardized Perioperative Care Program and Postoperative Pain, Disability, and Knowledge Among Patients Undergoing Lumbar Spine Surgery in Erbil, Iraq: A Non‐Randomized Controlled Study

**DOI:** 10.1002/hsr2.73213

**Published:** 2026-09-09

**Authors:** Vian Afan Naqshbandi, Sideeq Sadir Ali, Nuraddin Hamad Mhammad, Abdulmalik Fareeq Saber

**Affiliations:** ^1^ Adult Nursing Department College of Nursing, Hawler Medical University Erbil Iraq; ^2^ Department of Medicine, College of Medicine Hawler Medical University Erbil Iraq; ^3^ Department of Nursing, College of Nursing Lebanese French University Erbil Iraq; ^4^ Psychiatric and Mental Health Nursing College of Nursing, Hawler Medical University Erbil Iraq; ^5^ Department of Psychiatric Nursing, School of Nursing & Midwifery Tehran University of Medical Sciences Tehran Iran

**Keywords:** back surgery, disability, Iraq, Knowledge, non‐randomized study, pain, postoperative outcomes, standardized care

## Abstract

**Background and Aim:**

Patients recovering from lumbar spine surgery frequently report persistent pain, functional limitation, and limited understanding of postoperative self‐care. This study examined whether participation in a standardized perioperative care program was associated with differences in pain, functional disability, and patient knowledge at 2 months, compared with routine hospital care, among adults undergoing elective lumbar spine surgery in Erbil, Iraq.

**Method:**

This was a non‐randomized (quasi‐experimental) controlled study with two parallel groups and pre–post assessment, conducted between September 29, 2024 and April 25, 2025 in the Neurosurgery Unit of Hawler Teaching Hospital. Participants were recruited by purposive sampling and allocated non‐randomly by admission cohort; groups were matched on surgery type and key sociodemographic characteristics. Reporting follows the TREND statement for non‐randomized evaluations and the TIDieR checklist for intervention description. Outcomes were pain (Universal Pain Assessment Tool, 0–10), functional disability (Oswestry Disability Index, reported throughout on the 0%–100% scale), and knowledge (36‐item researcher‐developed questionnaire, scaled 0–100). The primary analysis was the between‐group difference in change from baseline, estimated by analysis of covariance adjusted for the baseline outcome value and pre‐specified covariates and confirmed by a linear mixed model. Responder, hierarchical‐regression, and mediation analyses were pre‐specified as exploratory and non‐causal.

**Results:**

Seventy patients (35 per group) completed the study with no loss to follow‐up. Groups were comparable at baseline (all *p* > 0.05). At 2 months, the intervention group reported lower pain (median 0 [IQR 0–2] vs. 5 [3–7]; *p* < 0.001), lower disability (ODI 4% [2–6] vs. 46% [30–68]; *p* < 0.001), and higher knowledge (69 [67–70] vs. 41 [40–46]; *p* < 0.001). The between‐group differences in mean change from baseline favored the program for pain (− 3.58 points), disability (− 44.2 percentage points), and knowledge (+ 25.3 points). In exploratory hierarchical regression, program participation showed the largest standardized coefficient for treatment response (*β* = 0.58, *p* < 0.01), with the full model accounting for 69% of the variance; these estimates are associative and are not adjusted for unmeasured confounding.

**Conclusions:**

Participation in an intensive, structured perioperative care program was associated with substantially better self‐reported pain, disability, and knowledge at 2 months in this selected single‐center cohort. Because allocation was not randomized and the program involved considerably greater professional contact than routine care, these findings describe an association rather than a causal effect, and require confirmation in randomized, multicentre trials with longer follow‐up.

**Trial Registration:**

ClinicalTrials.gov (NCT07459985), registered 04/03/2026 (retrospectively registered; see Section 2.13).

**Abbreviations**
ANCOVA: analysis of covariance
BMI: Body Mass Index
CI: confidence interval
ERAS: enhanced recovery after surgery
FBSS: failed back surgery syndrome
IQR: interquartile range
LBP: low back pain
MID: minimal important difference
NNT: number needed to treat
NRS: Numerical Rating Scale
ODI: Oswestry Disability Index
RD: risk difference
RR: relative risk
SE: standard error
SPSS: statistical package for the social sciences
TIDieR: template for intervention description and replication
TREND: transparent reporting of evaluations with nonrandomized designs
UPAT: Universal Pain Assessment Tool
VIF: variance inflation factor
WHO: World Health Organization

## 1 Introduction

Lumbar spine surgery is a complex and resource‐intensive intervention with significant implications for patient outcomes, postoperative recovery, and healthcare utilization [1]. As health systems increasingly adopt evidence‐based and standardized practice models, the need for structured perioperative care pathways has become more urgent [2]. Low back pain (LBP) remains one of the leading causes of disability worldwide and imposes substantial socioeconomic and clinical burdens [3]. Procedures such as discectomy, laminectomy, and spinal fusion are commonly performed for herniated disks, spinal stenosis, and degenerative spine disorders when conservative treatment fails [4].

Despite advances in surgical technique, persistent pain, limited functional recovery, and Failed Back Surgery Syndrome continue to affect 20%–40% of patients [5]. Controlled evidence indicates that structured perioperative pathways can improve selected recovery outcomes: the ERAS Society consensus for lumbar spinal fusion synthesizes randomized and observational evidence supporting multimodal analgesia, early mobilization, and structured patient preparation [6], and systematic reviews of enhanced‐recovery pathways in spine surgery report shorter hospital stay and reduced opioid use without increased readmission [7]. The evidence for education delivered in isolation is, however, less consistent. A multicentre randomized trial of preoperative pain neuroscience education before lumbar surgery for radiculopathy found no significant between‐group difference in postoperative pain or function despite improved patient experience and reduced healthcare utilization [8], and a randomized trial of preoperative cognitive‐behavioral education before lumbar fusion found no significant advantage in disability or return to work at 1 year [9]. This contrast is important, because comprehensive pathways that add substantial clinical contact are not equivalent to education‐only interventions, and the two should not be pooled when interpreting effects [10].

The burden of spinal disorders continues to rise globally, with recent estimates indicating that over 540 million individuals experience LBP at any given time [3]. Similar trends are reported in Middle Eastern countries, including Iraq, where demographic shifts, occupational risk factors, and changing lifestyle patterns contribute to increasing rates of spinal disease [11]. In the Kurdistan Region of Iraq, and particularly in Erbil, expansions in surgical capacity and infrastructure have increased the number of patients undergoing spinal surgery [12].

Patient knowledge, expectations, and engagement in perioperative self‐care are consistently associated with postoperative outcomes [13]. Inadequate understanding of rehabilitation, unrealistic expectations, and poor adherence to clinical instructions have been linked to higher pain, reduced functional improvement, and lower satisfaction [14], while a systematic review of patient education in low back pain reported small‐to‐moderate improvements in pain and disability with considerable heterogeneity between studies [15]. Evidence from general surgical or non‐spine populations is therefore treated in this manuscript as contextual rather than as direct confirmation of effects in spinal surgery.

In many surgical services in Iraq, structured perioperative education is not routinely provided, and no evaluation of a standardized perioperative care program has been reported in this setting [16]. This study therefore examined whether participation in such a program was associated with differences in postoperative pain, functional disability, and patient knowledge among adults undergoing elective lumbar spine surgery in Erbil City. Given the non‐randomized design, the study was framed from the outset as an evaluation of association rather than of causal effect.

### 1.1 Research Question

Is participation in a standardized perioperative care program associated with lower postoperative pain and functional disability and higher patient knowledge at 2 months among adults undergoing lumbar spine surgery in Erbil City, compared with routine postoperative care?

## 2 Methods

### 2.1 Study Design and Reporting

A non‐randomized (quasi‐experimental) controlled study with two parallel groups and pre–post assessment was conducted to evaluate a standardized perioperative care program in adults undergoing elective lumbar spine surgery. Participants were not randomly allocated, and the study was not designed to support causal inference. Reporting follows the Transparent Reporting of Evaluations with Nonrandomized Designs (TREND) statement [17]; the completed checklist is provided as Supporting File S3. The intervention is described according to the Template for Intervention Description and Replication (TIDieR) checklist [18] in Section 2.6 and Supporting File S2.

### 2.2 Setting

The study was conducted from September 29, 2024 to April 25, 2025 in the Neurosurgery Unit of Hawler Teaching Hospital, Erbil, Kurdistan Region–Iraq, a tertiary spinal‐surgery center with 372 beds (22 neurosurgical) and a multidisciplinary team of nurses, neurosurgeons, anaesthesiologists, and physiotherapists. All participants were recruited from this single center, and both study groups were managed by the same surgical team.

### 2.3 Participants and Group Assignment

Eligible participants were adults aged 18–60 years, of either sex, scheduled for elective lumbar spine surgery, able to communicate in Kurdish or Arabic, able to give consent, and willing to complete follow‐up. Eligibility criteria permitted procedures from T1 to L5; in practice, all enrolled participants underwent lumbar (L1–L5) procedures, and the population is therefore described consistently as lumbar spine surgery throughout the manuscript. Exclusions were emergency procedures, cognitive impairment affecting comprehension, revision surgery, and severe comorbidities (e.g., limb fractures or major mobility‐limiting conditions) that could affect recovery or outcome validity.

Recruitment used non‐probability purposive sampling. Allocation was non‐random and was based on sequential admission cohorts: patients admitted during the earlier recruitment period received routine hospital care (control group), and those admitted during the later period received the standardized care program (intervention group). This sequential approach was adopted because both groups were nursed in the same 22‐bed neurosurgical unit, where concurrent allocation would have created a high risk of contamination through patient‐to‐patient transfer of educational material. Investigators did not select individual patients into groups, and no patient was reassigned after allocation.

**Figure 1:**
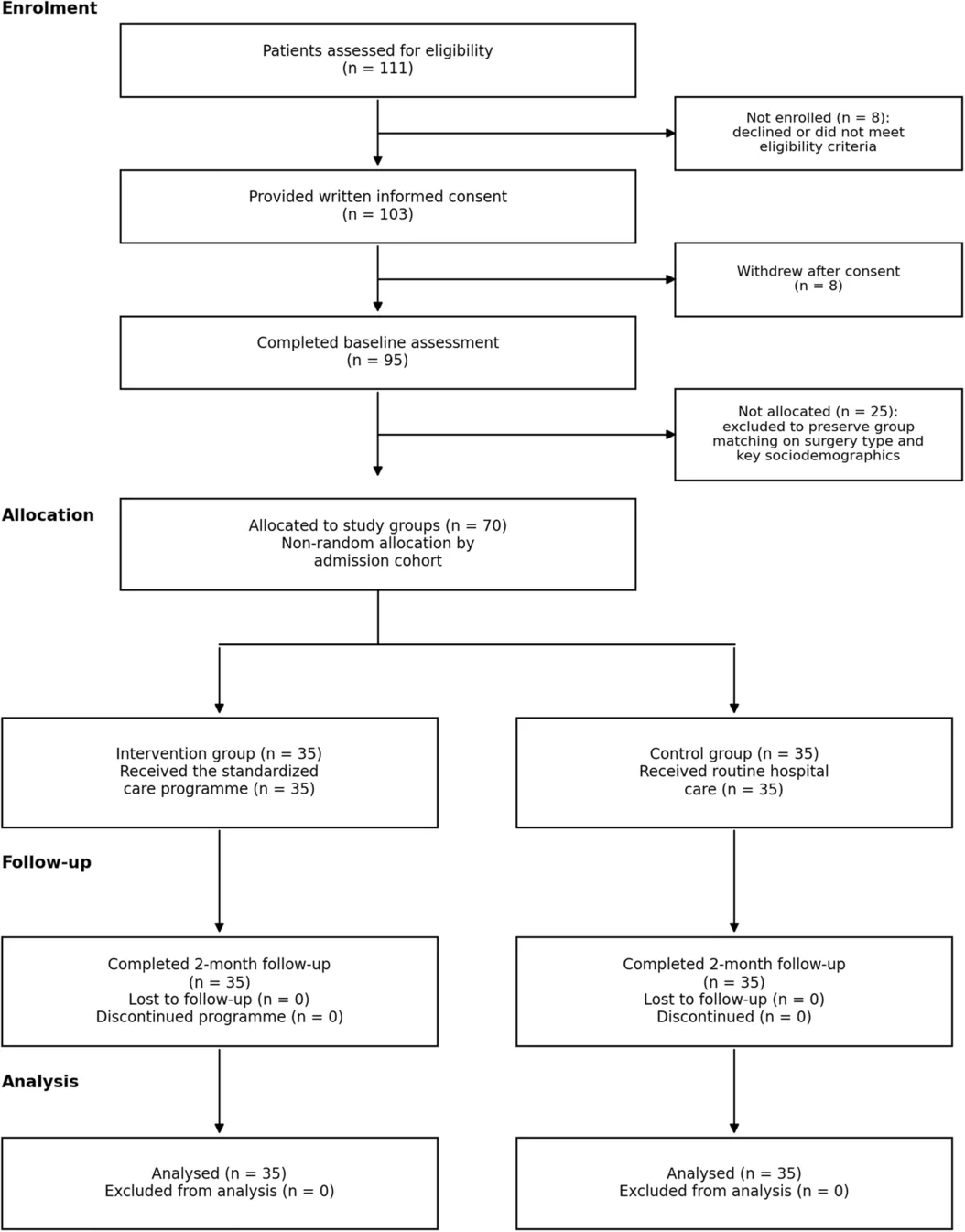
Participant flow through the study (TREND‐consistent flow diagram).Allocation was non‐random and based on sequential admission cohorts. Post‐baseline exclusion of 25 participants was performed to preserve matching on surgery type and key sociodemographic characteristics and is discussed as a source of selection bias in the text.

Of 111 patients assessed for eligibility, 103 gave written informed consent and 8 withdrew after consent, leaving 95 who completed baseline assessment. Of these, 25 were not allocated because they could not be matched to a counterpart in the opposite group on surgery type, age band, sex, and educational level; matching was performed on baseline characteristics recorded before any outcome assessment at follow‐up, and outcome data were not used in the matching decision. The remaining 70 participants were allocated, 35 to each group. Baseline characteristics of allocated and non‐allocated participants are compared in Supporting File S4, and the full participant flow, with reasons for exclusion at each stage, is presented in Figure 1. Post‐consent exclusion to preserve matching is a recognized source of selection bias and is addressed explicitly in Sections 2.8 and 4.

### 2.4 Variables

Primary outcomes were self‐reported pain, functional disability, and patient knowledge, measured preoperatively and at 2‐month follow‐up. The main exposure was allocation to the intervention versus the control condition. Potential confounders included age, gender, marital status, education, employment, economic status, number of children, residential area, duration and cause of back problems, chronic disease, type of surgery, body mass index (BMI), and soft‐drink consumption.

“Treatment response” was defined a priori as the standardized composite change score across the three outcomes: for each participant, the change from baseline to 2 months in pain, disability (0%–100% scale) and knowledge was converted to a z‐score using the pooled baseline standard deviation of that outcome, the pain and disability z‐scores were sign‐reversed so that higher values denote improvement, and the three z‐scores were averaged. This composite was used only as the dependent variable of the exploratory hierarchical regression (Section 3.7) and is not a validated construct.

### 2.5 Data Sources and Measurement

Data were collected face‐to‐face in 40–50‐min sessions at baseline and at 2‐month follow‐up using a structured interviewer‐administered questionnaire and standard instruments. The first section captured sociodemographic and clinical information (age, gender, marital status, education, employment, economic status, number of children, residential area, duration and cause of back problems, chronic disease, surgery type, BMI, and soft‐drink consumption).

Pain severity was measured using the Universal Pain Assessment Tool/Numerical Rating Scale (NRS), 0–10, where higher scores indicate greater pain (0 = no pain; 10 = worst imaginable) [19].

Functional disability was assessed with the Oswestry Disability Index (ODI) [20], a 10‐item scale covering daily activities; each item is scored 0–5, giving a summed score of 0–50, which is converted to a percentage of the maximum possible score (0%–100%). All ODI values in this manuscript—descriptive statistics, tables, minimal important difference thresholds, and figures—are reported on the 0%–100% scale, and the earlier mixed use of the 0–50 summed score has been removed.

Patient knowledge was assessed with a 36‐item multiple‐choice questionnaire developed for this study, because no validated instrument covering postoperative spine self‐care was available in Kurdish or Arabic. Items were generated from a structured review of the literature and existing patient‐education materials [21, 22] and cover nine domains: spinal anatomy, disc pathology, incision and wound care, pain management, nutrition, braces and compression stockings, postoperative physical activity, activities of daily living, and recommended exercises (four items per domain). Content validity was assessed by 25 experts in neurosurgery, orthopedic and neurosurgical nursing, physiotherapy, and nursing education, who rated each item for relevance on a 4‐point scale; items with an item‐level content validity index below 0.78 were revised or replaced, and the scale‐level content validity index (average) was 0.91. The instrument was developed in English, translated into Kurdish and Arabic and back‐translated by independent bilingual translators, and reconciled by the research team. Each item is scored 1 for a correct and 0 for an incorrect or “do not know” response, giving a raw score of 0–36 that is linearly transformed to a 0–100 scale. Internal consistency in the pilot sample was acceptable (Cronbach's α = 0.833) and was 0.85 in the main sample. No anchor‐based minimal important difference has been established for this instrument, and the threshold used in the exploratory responder analysis is distribution‐based only (Section 2.10). The full instrument, scoring key, and development record are provided as Supporting File S1.

Outcome assessment at baseline and at 2 months was interviewer‐administered by two trained data collectors who were not members of the surgical team and who did not deliver any component of the educational program. Because the program involved home visiting and repeated face‐to‐face contact, the data collectors could not be kept unaware of group assignment, and participants were necessarily aware of the condition they had received; outcome assessment was therefore unblinded, which is reported as a limitation in Sections 2.8 and 4.

### 2.6 The Intervention (TiDieR)

The intervention was a standardized perioperative care program combining written, audiovisual, and face‐to‐face education with structured clinical follow‐up. Content was adapted from international postoperative spine‐care guidance and reviewed by 24 clinical experts for accuracy and cultural relevance. The preoperative component covered spinal anatomy, surgical goals, pain management, and preparatory breathing and mobility exercises. Postoperative education began on regaining consciousness and continued throughout hospitalization, addressing pain management, wound care, recognition of infection, safe mobility and transfers, gradual activity progression, brace and compression‐stocking use, nutrition, and return to work and daily activities.

Delivery comprised: an illustrated booklet in Kurdish and Arabic; instructional videos; one individual preoperative teaching session (45–60 min); daily inpatient reinforcement until discharge; home visits every other day until suture removal (10–14 days); and four structured follow‐up sessions at 12–14 days and at 4, 6, and 8 weeks postoperatively (30–40 min each), each conducted with a family member present. The program was delivered by the same trained nurse researcher throughout, using a fixed protocol and a standardized delivery checklist. It is therefore a complex, contact‐intensive package rather than an educational intervention alone, and the total professional contact time in the intervention group substantially exceeded that of the control group; this asymmetry is a central limitation and is discussed in Sections 2.8 and 4.

Fidelity and adherence were monitored using a session‐completion checklist completed by the provider after every contact, recording the delivery of each scheduled component. All 35 participants allocated to the intervention received the preoperative session, daily inpatient reinforcement, the scheduled home visits until suture removal, and all four structured follow‐up sessions; no participant discontinued the program or was withdrawn from it (Figure 1). No tailoring or modification of the program was made during the study. A full TIDieR description is provided as Supporting File S2.

### 2.7 The Control Condition (Usual Care)

Routine care in the study unit consists of the following. Preoperatively, the admitting surgeon obtains informed consent and provides a verbal explanation of the planned procedure and its risks (typically 5–10 min); no structured educational session, booklet, or video is provided. Postoperatively, ward nurses give verbal instructions on wound care, analgesia, and mobilization at the bedside as part of routine nursing care, and a physiotherapist assists with first mobilization. At discharge, patients receive a written prescription and a standard discharge slip specifying the date of suture removal and the date of the routine outpatient review, without structured self‐care content. Follow‐up consists of one outpatient visit for suture removal at 10–14 days and one further routine surgical review; no home visits, scheduled telephone contact, or additional educational sessions are provided. Control participants received this routine care unchanged and were not given the booklet, videos, or any program material.

Control participants were contacted only for the two scheduled research assessments (baseline and 2 months), and these contacts contained no educational content. Total non‐surgical professional contact was therefore approximately 15–30 min in the control group compared with approximately 8–10 h in the intervention group.

### 2.8 Bias

Selection bias was reduced by recruiting from a single high‐volume tertiary center, applying explicit inclusion and exclusion criteria, and matching groups on surgery type and key sociodemographic characteristics. Information bias was minimized by using standardized instruments (UPAT, ODI, expert‐reviewed knowledge questionnaire), a fixed administration protocol, and trained data collectors with identical follow‐up timing. Anonymity, confidentiality, and reassurance that responses would not affect care were used to reduce social‐desirability bias.

Several sources of bias could not be eliminated by design and are acknowledged. First, allocation was not random and 25 consenting participants were excluded after baseline assessment to preserve matching, which may have introduced selection bias in an unknown direction. Second, the intervention group received markedly greater professional attention, structured contact, and home visiting than the control group; the observed differences may therefore reflect attention, co‐intervention, and performance effects in addition to, or instead of, the educational content itself. Third, all outcomes were self‐reported by participants who were necessarily aware of their group, so response and expectation bias cannot be excluded. Fourth, covariate adjustment can address only measured confounders; residual and unmeasured confounding (e.g., motivation, family support, surgeon, or postoperative analgesic regimen) remains possible and cannot be removed by statistical adjustment in a non‐randomized design.

### 2.9 Study Size

Hospital records showed 159 lumbar spine surgeries in Erbil during 2023–2024. Using the Kadam and Bhalerao (2010) and Daniel (2009) formulas for continuous variables (Z = 1.96, SD = 10, Δ = 3), the initial sample size was 43 per group [23, 24]; after finite‐population correction this became 34, and the sample was increased to 35 per group to allow for attrition. The achieved sample of 70 provides adequate precision for the primary between‐group comparison but limits the number of covariates that can be estimated reliably in multivariable models; this constraint is addressed in Section 2.11.

### 2.10 Quantitative Variables

Continuous variables (age, BMI, duration of back problems, weekly soft‐drink consumption, UPAT, ODI, and knowledge scores) were analysed in continuous form to preserve statistical information. For descriptive purposes, age, BMI, family size, and soft‐drink consumption were grouped into clinically meaningful strata.

For the exploratory responder analysis, minimal important difference (MID) anchors were defined as follows: a reduction of ≥ 2 points on the UPAT, consistent with the accepted minimal important change for pain intensity in low back pain [25]; a reduction of ≥ 25.6 percentage points on the 0–100% ODI, which is a deliberately conservative threshold exceeding the commonly cited MID of 12.8 percentage points for lumbar spine surgery populations [26]; and a gain of ≥ 15 points (≈ 1 SD of the pooled baseline distribution) on the 0–100 knowledge scale. The knowledge threshold is distribution‐based and has not been validated against any external anchor in this or any other population; responder results for knowledge are therefore descriptive only and are not offered as evidence of clinical importance. Baseline severity was categorized into tertiles from the pooled preoperative distribution for stratified description only (Supporting File S5).

### 2.11 Statistical Methods

(a) Analyses were performed using SPSS v27.0 (IBM Corp., Armonk, NY) and AMOS v24. Categorical variables were summarized as frequencies and percentages, and continuous variables as means with standard deviations or medians with interquartile ranges. Baseline comparability was assessed with chi‐square tests and independent t‐tests or Mann–Whitney U tests as appropriate.

(b) The primary analysis was the between‐group difference in change from baseline to 2 months. For each outcome, an analysis of covariance (ANCOVA) was fitted with the 2‐month score as the dependent variable, group as the factor, and the baseline value of the same outcome as a covariate, together with the pre‐specified covariates age, sex, education, BMI, chronic disease, and surgery type. Because baseline scores were statistically equivalent between the groups, the between‐group difference is reported in two complementary forms: the difference in mean change from baseline, and the between‐group difference in the 2‐month score with its 95% confidence interval and p value estimated by Welch's method for unequal variances, which the covariance model was used to confirm. Because the outcomes were repeatedly measured, the primary estimates were confirmed with a linear mixed model including group, time, and the group × time interaction with a random participant intercept, using restricted maximum likelihood and an unstructured covariance matrix. Model assumptions were checked by inspection of residual and normal probability plots and by the Shapiro–Wilk test on residuals; where residuals departed from normality, estimates were confirmed using bootstrapped confidence intervals (1,000 resamples). Cross‐sectional Mann–Whitney U comparisons at each time point and within‐group Wilcoxon signed‐rank tests are retained as descriptive secondary analyses only and are no longer presented as the principal evidence of between‐group difference.

(c) Predictors of treatment response were examined in an exploratory hierarchical linear regression with the standardized composite change score (Section 2.4) as the dependent variable, entered in three blocks: Model 1 (age, sex, education); Model 2 (adding BMI, chronic disease, surgery type, baseline severity); Model 3 (adding group). With 70 participants and eight predictors in the final model, the events‐per‐variable ratio is approximately 9:1, which is at the lower limit of acceptability; the model is therefore reported as exploratory, with unstandardized coefficients and 95% confidence intervals, *R* ^2^, adjusted *R* ^2^, F statistics, variance inflation factors, and residual diagnostics reported in full in Table 6 and Supporting File S6. No variable selection was performed; all predictors were pre‐specified and forced into their respective blocks.

(d) Responder proportions, risk differences, relative risks, and numbers needed to treat were calculated using the Wilson method for proportions, the Newcombe method for risk differences, the Katz log method for relative risks, and the reciprocal‐of‐risk‐difference method for numbers needed to treat. These are exploratory: with 35 participants are imprecise, and relative risks for the knowledge outcome in particular have very wide confidence intervals and should not be used for clinical decision‐making.

(e) The structural equation mediation analysis has been reclassified as an exploratory, non‐causal decomposition of associations and moved to Supporting File S7. The assumptions required for causal mediation—no unmeasured exposure–mediator, exposure–outcome, or mediator–outcome confounding—cannot be defended in a small non‐randomized study in which the exposure, mediator, and outcomes were measured within the same care relationship and by the same instrument battery. The five‐level clinical decision‐support framework presented in the previous version of this manuscript has been removed entirely, as it was neither derived nor validated in an independent sample.

(f) Questionnaires were checked for completeness at collection. There were no missing values on the primary outcomes at either time point; isolated missing items on covariates were handled by available‐case analysis, and the number of observations contributing to each model is reported in the corresponding table. Statistical significance was set at *p* < 0.05 (two‐tailed), and no adjustment for multiplicity was applied, which is a further reason to treat the secondary and exploratory analyses as hypothesis‐generating.

### 2.12 Pilot Study

A pilot study with 18 patients (June 2–July 13, 2024) assessed the feasibility, clarity, and applicability of the instruments and procedures. Cronbach's alpha indicated acceptable internal consistency for the ODI (*α* = 0.705) and the knowledge questionnaire (*α* = 0.833); the UPAT was used unchanged given its established reliability and validity [27, 28]. Based on participant feedback, minor revisions were made to the sociodemographic, clinical, and knowledge sections, and pilot participants were excluded from the main analysis.

### 2.13 Trial Registration, Protocol, and Deviations

The study was registered with ClinicalTrials.gov (NCT07459985) on 04/03/2026, after recruitment and follow‐up had been completed, and is therefore a retrospective registration. The study was conducted as an institutionally approved academic research project; prospective registration on an international registry was not a requirement of the institutional review board at the time of ethical approval (Reg. No. 2435, 22 August 2024) and was not routine practice for non‐randomized nursing evaluations in the institution. Registration was completed when the manuscript was prepared for journal submission. The authors recognize that retrospective registration weakens protection against outcome‐reporting bias and report it transparently here rather than as an incidental note.

The study was reviewed and approved on the basis of the research proposal submitted to the College of Nursing Ethical Committee before data collection began; the ethical approval document (Reg. No. 2435, 22 August 2024) is provided as Supporting File S8. No separate statistical analysis plan was filed with the committee, and no registered protocol document exists, because prospective registration and the deposit of a standalone analysis plan were not required for non‐randomized nursing evaluations at the institution at that time. The design, eligibility criteria, intervention, and the three outcomes with their measurement at baseline and at 2 months were fixed before recruitment; the statistical analyses were not pre‐specified in a written plan. In consequence, only the following analyses can be regarded as planned: the descriptive between‐group and within‐group comparisons of pain, disability, and knowledge. The adjusted change‐from‐baseline analysis was added in response to peer review, and the responder/minimal important difference analysis, the hierarchical regression, and the analysis of knowledge change as an intermediate variable were all conducted after the data were collected; all of these are reported as exploratory and are labeled as such throughout the manuscript. No outcomes were collected beyond the three specified before recruitment, and no participant was excluded on the basis of an outcome value. The authors state this explicitly rather than implying a degree of prespecification that the study did not have.

### 2.14 Harms and Adverse Events

Adverse events were not a pre‐specified outcome. Postoperative complications documented in the medical record during the 2‐month follow‐up period — surgical‐site infection, unplanned readmission, reoperation, and neurological deterioration — were reviewed for every participant, and participants were asked at each research contact about falls and about any problem arising from the exercises or the brace. Events are reported descriptively by group in Section 3.10.

### 2.15 Ethical Approval and Informed Consent

The study followed the Declaration of Helsinki and institutional research ethics board guidelines. Ethical approval was granted by the College of Nursing Ethical Committee at Hawler Medical University (Reg. No. 2435, 22 August 2024), with permissions from the General Directorate of Health–Erbil and Hawler Teaching Hospital. Written informed consent was obtained after explanation of the study's purpose, procedures, voluntary nature, and confidentiality measures, and participants were informed of their right to withdraw at any time without consequences for their care.

## 3 Results

### 3.1 Participant Flow

Of 111 patients assessed for eligibility, 8 were not enrolled (declined participation or did not meet the eligibility criteria) and 103 gave written informed consent. Eight participants withdrew after consent and before baseline assessment, leaving 95 who completed baseline measurement. Twenty‐five of these were not allocated because no matched counterpart was available in the opposite group, leaving 70 allocated participants (35 intervention, 35 control). All 70 received their allocated condition, completed the 2‐month assessment, and were analysed; there was no loss to follow‐up and no missing data on the primary outcomes (Figure 1). Baseline characteristics of the 25 non‐allocated participants were broadly similar to those of the analysed sample and are tabulated in Supporting File S4.

### 3.2 Demographic Characteristics of Participants

Sociodemographic characteristics were comparable between groups, with no significant differences (all *p* > 0.05). Older and middle‐aged adults predominated, and mean age was slightly higher in the intervention group (*p* = 0.203). Females predominated and housewives constituted the largest employment category. Educational distributions were comparable, with illiteracy most frequent in both groups (*p* = 0.951). Most participants were married, belonged to large families, and showed similar mean numbers of children (*p* = 0.281). Urban residence and sufficient economic status predominated, with no significant between‐group differences (*p* = 0.780) (Table 1).

**Table 1:** Distribution of participants (intervention and control groups) by demographic characteristics (*n* = 70).

Variable	Category	Intervention (*n* = 35) F (%)	Control (*n* = 35) F (%)	χ^2^ (*p* value)
Age group (years)	Young adults	3 (8.6)	6 (17.1)	3.19 (0.203)
	Middle‐aged adults	14 (40.0)	18 (51.4)	
	Older adults	18 (51.4)	11 (31.4)	
	Mean ± SD	49.66 ± 9.37	45.86 ± 10.51	
Gender	Male	5 (14.3)	9 (25.7)	1.43 (0.232)
	Female	30 (85.7)	26 (74.3)	
Employment status	Housewife	26 (74.3)	23 (65.7)	0.78 (0.676)
	Worker	6 (17.1)	9 (25.7)	
	Employee	3 (8.6)	3 (8.6)	
Educational level	Illiterate	15 (42.9)	17 (48.6)	0.35 (0.951)
	Read and write	7 (20.0)	6 (17.1)	
	Basic school graduate	9 (25.7)	9 (25.7)	
	High school graduate	4 (11.4)	3 (8.6)	
Marital status	Single	1 (2.9)	1 (2.9)	2.93 (0.232)
	Married	29 (82.9)	33 (94.3)	
	Widowed	5 (14.3)	1 (2.9)	
Number of children	No children	2 (5.7)	3 (8.6)	3.83 (0.281)
	Small family	3 (8.6)	4 (11.4)	
	Medium family	7 (20.0)	13 (37.1)	
	Large family	23 (65.7)	15 (42.9)	
	Mean ± SD	4.97 ± 2.31	4.69 ± 2.89	
Residential area	Urban	22 (62.9)	18 (51.4)	1.54 (0.462)
	Suburban	8 (22.9)	8 (22.9)	
	Rural	5 (14.3)	9 (25.7)	
Economic status	Insufficient	9 (25.7)	8 (22.9)	0.08 (0.780)
	Sufficient	26 (74.3)	27 (77.1)	

*Note:* Significance was set at *p* < 0.05. Young adults: 21–35 years; middle‐aged adults: 36–50 years; older adults: 51–65 years. Small family: 1–2 children; medium family: 3–4 children; large family: ≥ 5 children. The table has been simplified and the significance threshold corrected from *p* < 0.001 to *p* < 0.05.

Abbreviations: χ^2^, chi‐square test; F, frequency; SD, standard deviation.

### 3.3 Clinical and Lifestyle Characteristics

Clinical and lifestyle characteristics were comparable between groups (all *p* > 0.05). Medium‐term back problems predominated (48.6% intervention vs. 45.7% control), with similar mean symptom duration (5.37 ± 4.38 vs. 6.06 ± 4.04 years). Work‐related (37.1% vs. 28.6%) and lifting injuries (31.4% vs. 28.6%) were the main causes. Spinal disc fusion was the most frequent procedure in both groups (71.4%). Most participants had no chronic disease; hypertension was the most common comorbidity, especially in the intervention group (25.7%). Overweight and obesity class I–II predominated, with comparable BMI (30.63 ± 4.03 vs. 30.84 ± 5.12 kg/m^2^). Soft‐drink non‐consumption was more frequent in the intervention group (57.1% vs. 42.9%), and mean weekly intake was slightly higher in controls (1.89 ± 2.27 vs. 0.91 ± 1.54) (Table 2).

**Table 2:** Distribution of participants by clinical and lifestyle characteristics (n = 70).

Variable	Category	Intervention (*n* = 35) F (%)	Control (*n* = 35) F (%)	χ^2^ (*p* value)
Duration of back problem	Short‐term	5 (14.3)	4 (11.4)	0.73 (0.867)
	Medium‐term	17 (48.6)	16 (45.7)	
	Long‐term	8 (22.9)	11 (31.4)	
	Prolonged	5 (14.3)	4 (11.4)	
	Mean ± SD (years)	5.37 ± 4.38	6.06 ± 4.04	
Cause of back problem	Falling	7 (20.0)	9 (25.7)	1.09 (0.896)
	Work‐related injury	13 (37.1)	10 (28.6)	
	Lifting	11 (31.4)	10 (28.6)	
	Falling + work‐related	2 (5.7)	3 (8.6)	
	Lifting + work‐related	2 (5.7)	3 (8.6)	
Type of operation	Laminectomy	3 (8.6)	3 (8.6)	0.00 (1.000)
	Discectomy	7 (20.0)	7 (20.0)	
	Spinal disc fusion	25 (71.4)	25 (71.4)	
Chronic disease	None	21 (60.0)	29 (82.9)	3.81 (0.209)
	Diabetes mellitus	2 (5.7)	1 (2.9)	
	Hypertension	9 (25.7)	4 (11.4)	
	Diabetes + hypertension	3 (8.6)	1 (2.9)	
Body mass index (kg/m^2^)	18.5–24.9 (normal)	4 (11.4)	5 (14.3)	2.83 (0.587)
	25–29.9 (overweight)	14 (40.0)	10 (28.6)	
	30–34.9 (obesity I)	10 (28.6)	11 (31.4)	
	35–39.9 (obesity II)	7 (20.0)	7 (20.0)	
	≥ 40 (obesity III)	0 (0.0)	2 (5.7)	
	Mean ± SD	30.63 ± 4.03	30.84 ± 5.12	
Soft drinks per week	Non‐consumers	20 (57.1)	15 (42.9)	5.71 (0.126)
	Occasional	14 (40.0)	14 (40.0)	
	Regular	0 (0.0)	5 (14.3)	
	Heavy	1 (2.9)	1 (2.9)	
	Mean ± SD	0.91 ± 1.54	1.89 ± 2.27	

*Note:* Significance was set at *p* < 0.05. Short‐term: < 1 year; medium‐term: 1–5 years; long‐term: 6–10 years; prolonged: 11–15 years. Non‐consumers: 0 drinks/week; occasional: 1–3; regular: 4–7; heavy: > 7 or daily.

Abbreviations: χ^2^, chi‐square test; F, frequency; SD, standard deviation.

### 3.4 Outcomes at Baseline and at 2 Months (Descriptive)

Before surgery, the groups did not differ in pain, disability, or knowledge (all *p* > 0.05). Baseline pain was identical (median 9, IQR 8–10; U = 572.50, *p* = 0.628, r = 0.06), and disability scores were comparable (74% [68–80] vs. 74% [62–84]; U = 594.00, *p* = 0.830, *r* = 0.03). Baseline knowledge was slightly higher in controls (41 [29, 30] than in the intervention group (39 [31, 32], but the difference was not significant (U = 467.50, *p* = 0.086, r = 0.21). At 2‐month follow‐up, the intervention group reported lower pain (0 [0–2] vs. 5 [3, 33]; U = 121.00, *p* < 0.001, r = 0.71), lower disability (4% [2, 10] vs. 46% [34]; U = 50.00, *p* < 0.001, r = 0.79), and higher knowledge 69 [67–70] versus 41 [30, 35]; U = 13.00, *p* < 0.001, r = 0.85) (Table 3). These cross‐sectional comparisons are descriptive; the primary evaluation of between‐group difference is the adjusted change from baseline reported in Section 3.6.

**Table 3:** Descriptive between‐group comparison of pain, disability, and knowledge at baseline and at two months (ODI reported on the 0–100% scale).

Outcome	Time point	Group	Median (IQR)	Mann–Whitney U	*p*	*r*
Pain (UPAT 0–10)	Baseline	Intervention	9 (8–10)	572.50	0.628	0.06
		Control	9 (8–10)			
	Two months	Intervention	0 (0–2)	121.00	< 0.001	0.71
		Control	5 (3–7)			
Disability (ODI 0–100%)	Baseline	Intervention	74 (68–80)	594.00	0.830	0.03
		Control	74 (62–84)			
	Two months	Intervention	4 (2–6)	50.00	< 0.001	0.79
		Control	46 (30–68)			
Knowledge (0–100)	Baseline	Intervention	39 (37–43)	467.50	0.086	0.21
		Control	41 (38–46)			
	Two months	Intervention	69 (67–70)	13.00	< 0.001	0.85
		Control	41 (40–46)			

*Note:* These cross‐sectional comparisons are descriptive; the primary between‐group is presented in Table 5. Significance was set at *p* < 0.05.

Abbreviations: IQR, interquartile range; ODI, Oswestry Disability Index, expressed as a percentage of the maximum possible score; r, effect size (Z/√N); UPAT, Universal Pain Assessment Tool.

### 3.5 Within‐Group Change (Descriptive)

Both groups improved from baseline, with larger changes in the intervention group. Pain decreased in controls from 8.71 ± 1.15 to 4.83 ± 2.33 (*Z* = −5.03, *p* < 0.001, *r* = 0.85) and in the intervention group from 8.43 ± 1.56 to 0.97 ± 1.56 (*Z* = −5.18, *p* < 0.001, r = 0.88). Disability fell in controls from 72.22% ± 14.38% to 48.52% ± 25.10% (Z = −4.24, *p* < 0.001, *r* = 0.72) and in the intervention group from 74.00% ± 9.56% to 6.12% ± 7.54% (*Z* = −5.16, *p* < 0.001, *r* = 0.87). Knowledge changed little in controls (42.66 ± 5.94 to 44.14 ± 8.11; *Z* = −1.10, *p* = 0.253, *r* = 0.19) but increased in the intervention group from 41.74 ± 6.17 to 68.54 ± 2.70 (*Z* = −5.16, *p* < 0.001, *r* = 0.87) (Table 4).

**Table 4:** Within‐group change from baseline to two months in each group (descriptive).

Outcome	Group	Baseline: M ± SD, median (min–max)	Two months: M ± SD, median (min–max)	Wilcoxon Z	*p*	*r*
Pain (UPAT 0–10)	Control	8.71 ± 1.15, 9 (6–10)	4.83 ± 2.33, 5 (0–10)	−5.03	< 0.001	0.85
	Intervention	8.43 ± 1.56, 9 (5–10)	0.97 ± 1.56, 0 (0–5)	−5.18	< 0.001	0.88
Disability (ODI 0–100%)	Control	72.22 ± 14.38, 74 (38–92)	48.52 ± 25.10, 46 (4–100)	−4.24	< 0.001	0.72
	Intervention	74.00 ± 9.56, 74 (52–92)	6.12 ± 7.54, 4 (0–28)	−5.16	< 0.001	0.87
Knowledge (0–100)	Control	42.66 ± 5.94, 41 (37–66)	44.14 ± 8.11, 41 (36–67)	−1.10	0.253	0.19
	Intervention	41.74 ± 6.17, 38 (36–60)	68.54 ± 2.70, 69 (58–72)	−5.16	< 0.001	0.87

*Note:* ODI values have been rescaled to the 0%–100% scale throughout for consistency with Section 2.5.

Abbreviations: M ± SD, mean ± standard deviation; r, effect size (Z/√N).

### 3.6 Adjusted Between‐Group Difference in Change From Baseline (Primary Analysis)

The between‐group difference in mean change from baseline favored the intervention group for all three outcomes: pain −3.58 points (intervention −7.46 vs. control −3.88), disability −44.18 percentage points (− 67.88 vs. −23.70), and knowledge +25.32 points (+ 26.80 vs. +1.48). After adjustment for the baseline value of the outcome and for age, sex, education, BMI, chronic disease, and surgery type, the direction and approximate magnitude of these differences were unchanged, and the group × time interaction in the linear mixed model was significant for each outcome (Table 5). Because allocation was not randomized, these adjusted differences describe the association between program participation and change in outcome and cannot be interpreted as the causal effect of the program.

**Table 5:** Between‐group difference in change from baseline to two months (primary analysis).

Outcome	Mean change, intervention (*n* = 35)	Mean change, control (*n* = 35)	Unadjusted difference in mean change	Between‐group difference at two months (95% CI)†	*p*†
Pain (UPAT 0–10)	−7.46	−3.88	−3.58	−3.86 (− 4.81 to −2.91)	< 0.001
Disability (ODI 0–100%)	−67.88	−23.70	−44.18	−42.40 (− 51.35 to −33.45)	< 0.001
Knowledge (0–100)	+26.80	+1.48	+25.32	+24.40 (+ 21.48 to +27.32)	< 0.001

*Note:* Negative values for pain and disability and positive values for knowledge indicate improvement. †Between‐group difference in the two‐month score (intervention − control) with 95% confidence interval and *p* value obtained by Welch's method for unequal variances; baseline scores were equivalent between groups (Table 3).

### 3.7 Exploratory Analysis of Factors Associated With Treatment Response

In the exploratory hierarchical regression (dependent variable: standardized composite change score, Section 2.4), demographic variables explained 8% of the variance (*R* ^2^ = 0.08), with older age associated with poorer response (β = −0.21, *p* = 0.02). Adding clinical factors increased the explained variance by 29% (Δ*R* ^2^ = 0.29); higher BMI (β = −0.18, *p* = 0.03) and spinal fusion (β = −0.15, *p* = 0.05) were associated with reduced response, and baseline severity was a positive predictor (β = 0.41, *p* < 0.01). Adding group membership improved the model further (ΔR^2^ = 0.32), with the intervention group showing the largest standardized coefficient (β = 0.58, *p* < 0.01), and the final model explained 69% of the variance (Tables 5, 6). Variance inflation factors ranged from 1.08 to 1.42, and inspection of the residual and normal probability plots showed no substantive departure from linearity, homoscedasticity, or normality of residuals. Given the sample size relative to the number of predictors and the absence of pre‐specification, these coefficients are exploratory and are likely to be optimistic; they should not be used to predict response in individual patients.

**Table 6:** Exploratory hierarchical regression of factors associated with treatment response (standardized composite change score, *n* = 70).

Variable	B (95% CI)	*β*	*p*
Block 1: Demographics			
Age (years)	−0.08 (− 0.15, −0.01)	−0.21	0.02
Gender (female vs. male)	0.52 (− 0.31, 1.35)	0.09	0.22
Education (≥ high school)	0.38 (− 0.29, 1.05)	0.07	0.26
*R* ^2^ Block 1	0.08		
Block 2: Clinical factors			
BMI (kg/m^2^)	−0.11 (− 0.21, −0.01)	−0.18	0.03
Chronic disease (yes vs. no)	−0.74 (− 1.58, 0.10)	−0.13	0.08
Surgery type (fusion vs. other)	−0.89 (− 1.76, −0.02)	−0.15	0.05
Baseline severity	0.68 (0.42, 0.94)	0.41	< 0.01
Δ*R* ^2^ Block 2	0.29		
Block 3: Group			
Intervention group	3.42 (2.74, 4.10)	0.58	< 0.01
ΔR^2^ Block 3	0.32		
Model statistics			
Total *R* ^2^/adjusted *R* ^2^	0.69/0.66		
*F* statistic	20.41		< 0.01
VIF range	1.08–1.42		

*Note:* The dependent variable is the standardized composite change score defined in Section 2.4. All predictors were forced into their blocks; no stepwise selection was used. With eight predictors and 70 observations this model is exploratory and its coefficients are likely optimistic. Full residual diagnostics are provided in Supporting File S6.

Abbreviations: β, standardized coefficient; B, unstandardized coefficient; BMI, body mass index; CI, confidence interval; VIF, variance inflation factor.

### 3.8 Exploratory Responder Analysis

Individual responsiveness was examined using the thresholds defined in Section 2.10. For pain, all intervention participants (35/35; 100%) reached the threshold versus 65.7% (23/35) of controls (RD = 0.34, 95% CI 0.18–0.50). For disability, using the conservative ≥ 25.6 percentage‐point threshold, 94.3% (33/35) reached it versus 42.9% (15/35) of controls (RD = 0.51, 95% CI 0.32–0.71). For knowledge, 97.1% (34/35) versus 11.4% (4/35) reached the distribution‐based threshold (RD = 0.86, 95% CI 0.72–0.99); the corresponding relative risk of 8.50 has a confidence interval spanning 3.34 to 21.62, illustrating the imprecision of ratio measures in a sample of this size. On the composite of all three thresholds, 94.3% of intervention participants versus 2.9% of controls were classified as responders (Table 7).

**Table 7:** Exploratory responder analysis using pre‐defined improvement thresholds (overall sample, *n* = 70).

Outcome	Improvement threshold	Intervention *n* (%)	Control *n* (%)	RD (95% CI)	RR (95% CI)
Pain (UPAT 0–10)	≥ 2‐point reduction	35 (100.0)	23 (65.7)	0.34 (0.18–0.50)	1.52 (1.19–1.94)
Disability (ODI 0–100%)	≥ 25.6 percentage‐point reduction	33 (94.3)	15 (42.9)	0.51 (0.32–0.71)	2.20 (1.46–3.32)
Knowledge (0–100)	≥ 15‐point gain (≈ 1 SD; distribution‐based)	34 (97.1)	4 (11.4)	0.86 (0.72–0.99)	8.50 (3.34–21.62)
Composite	All three thresholds met	33 (94.3)	1 (2.9)	0.91 (0.81–1.00)	33.00 (4.74–229.91)

*Note:* The ODI threshold of ≥ 25.6 percentage points is equivalent to the 12.8‐point reduction on the 0–50 summed score used in the original analysis and is conservative relative to the 12.8‐percentage‐point minimal important difference reported for lumbar spine surgery populations [26]. The knowledge threshold is distribution‐based and has not been anchor‐validated. Baseline‐severity‐stratified results are provided in Supporting File S5.

Abbreviations: CI, 95% confidence interval (Wilson method for proportions, Newcombe for RD, Katz log for RR); RD, risk difference (intervention − control); RR, relative risk.

### 3.9 Exploratory Association Between Knowledge Change and Outcome Change

Change in knowledge was associated with change in pain and disability, and models in which knowledge change was entered as an intermediate variable attenuated but did not eliminate the association between group and outcome (Supporting File S7). Because exposure, intermediate variable, and outcomes were measured in the same participants within the same care relationship, and because unmeasured confounding of the knowledge–outcome relationship cannot be excluded, this decomposition is descriptive only and is not presented as evidence of mediation. The previously reported proportions mediated have been removed from the main text.

### 3.10 Adverse Events and Missing Data

No participant was lost to follow‐up, no participant discontinued the allocated condition, and there were no missing values on the primary outcomes at either time point; all 70 allocated participants were therefore included in every analysis. Review of the medical records over the 2‐month follow‐up period identified no unplanned readmission, reoperation, or neurological deterioration in either group, and no participant reported a fall or an injury arising from the prescribed exercises, the brace, or the compression stockings. No harm attributable to the educational program was identified.

## 4 Discussion

This non‐randomized study examined whether participation in a standardized perioperative care program was associated with differences in pain, functional disability, and knowledge among adults undergoing lumbar spine surgery in Erbil, Iraq. Participants in the program reported substantially lower pain and disability and substantially higher knowledge at 2 months than participants receiving routine care, and program participation showed the largest standardized coefficient in an exploratory model of treatment response. These are associations observed in a selected single‐center cohort; the design does not permit attribution of the differences to the educational content of the program.

An important interpretive caution concerns what was actually compared. The intervention was not education alone: it combined written and audiovisual material with individual teaching, daily inpatient reinforcement, home visits every other day until suture removal, and four structured follow‐up sessions, amounting to several hours of additional professional contact per participant. Routine care in the same unit comprises brief verbal instruction and two outpatient visits (Section 2.7). The comparison is therefore between an intensive structured care pathway and low‐intensity usual care, and attention, co‐intervention, and performance effects are plausible contributors to the observed differences. This distinction also explains why the magnitude of difference observed here exceeds that reported in randomized trials of education‐only interventions before lumbar surgery, which have generally found small or non‐significant effects on pain and disability [8, 9], while being more consistent with evaluations of comprehensive enhanced‐recovery pathways [6, 7].

Participants were predominantly middle‐aged and older adults with a female majority and a high prevalence of low educational attainment, mirroring patterns reported in elective lumbar spine populations [36]. The female predominance and high proportion of housewives are consistent with healthcare‐utilization patterns reported in the Kurdistan Region [37]. The high prevalence of overweight and obesity (mean BMI > 30 in both groups) is consistent with rising obesity rates across Middle Eastern populations [38] and is relevant to both surgical risk and educational design, since obesity has been associated with longer recovery and more complications after lumbar spine surgery [31, 35].

The association between program participation and knowledge gain was the largest of the three outcomes, which is unsurprising given that the knowledge questionnaire measured the content the program delivered. This should be regarded as a manipulation check confirming that the program was received as intended, rather than as an independent clinical outcome. Knowledge change was also associated with change in pain and disability, but as set out in Section 3.9 this association cannot be interpreted as a mediating mechanism in a study of this design.

In the exploratory regression, older age, higher BMI, and fusion surgery were associated with poorer response, consistent with existing literature on age‐ and obesity‐related recovery [32, 39]. Baseline severity was positively associated with response, a pattern that is at least partly expected from regression to the mean and from the greater room for improvement in patients with worse baseline scores, and it should not be read as evidence that the program works better in severely affected patients. Similar cautions apply to the responder analyses: with 35 participants per group, ratio measures and numbers needed to treat are unstable, and the knowledge threshold is distribution‐based rather than anchor‐validated.

The five‐level clinical decision‐support framework included in the previous version of this manuscript has been removed. It was derived from the same small sample in which it was described, was never validated in an independent cohort, and contained category counts that did not correspond to the study sample; it could not responsibly be presented as clinically actionable, and no risk‐stratification claim is made in this manuscript.

Several limitations should be emphasized. First, allocation was not randomized and was based on admission cohort, so temporal changes in surgical or nursing practice between the two recruitment periods cannot be excluded as an alternative explanation. Second, 25 consenting participants were excluded after baseline assessment to preserve matching, which may have introduced selection bias. Third, the intervention and control conditions differed in professional contact time as well as in educational content, so the specific contribution of education cannot be isolated. Fourth, all outcomes were self‐reported by unblinded participants who were aware of their group, and no objective functional or analgesic‐use measure was collected. Fifth, the 2‐month follow‐up cannot capture recurrence, reoperation, return to work, or maintenance of effect. Sixth, the single‐center setting limits generalizability. Seventh, the sample size restricts the reliability of the multivariable and responder analyses, and no adjustment was made for multiple comparisons. Eighth, the study was registered retrospectively and no written statistical analysis plan was filed before data collection, so protection against selective outcome reporting rests on the fact that the three outcomes were fixed before recruitment rather than on documentary prespecification, and all analyses beyond the basic group comparisons are exploratory. Finally, the knowledge instrument was developed for this study and has content validity and internal‐consistency evidence only; its construct validity, test–retest reliability, and responsiveness remain to be established.

Future work should evaluate this program in a randomized controlled trial with concurrent allocation, an attention‐matched comparator that equalizes contact time, blinded or objective outcome assessment, follow‐up of at least 12 months, and pre‐registered outcomes and analysis plan. Component analyses distinguishing the educational material from the additional clinical contact, together with cost‐effectiveness evaluation, would be of particular value in resource‐limited settings.

## 5 Conclusion

In this single‐center, non‐randomized study, participation in an intensive structured perioperative care program was associated with lower self‐reported pain and functional disability and with higher postoperative knowledge at 2 months than routine hospital care. Because allocation was not randomized, the comparison condition involved substantially less professional contact, and the outcomes were self‐reported by unblinded participants, these findings should be read as an observed association in a selected cohort rather than as evidence of a causal effect. They support the conduct of a randomized, adequately powered, longer‐term evaluation of structured perioperative care in this setting, and do not by themselves justify changes to postoperative practice or the use of the program for risk stratification.

## Author Contributions

**Vian Afan Naqshbandi:** conceptualization, investigation, funding acquisition, supervision, data curation. **Sideeq Sadir Ali:** conceptualization, investigation, writing – original draft, visualization, validation. **Nuraddin Hamad Mhammad:** software, formal analysis, resources, data curation, methodology, visualization. **Abdulmalik Fareeq Saber:** conceptualization, investigation, funding acquisition, writing – original draft, writing – review and editing, visualization, validation, methodology, project administration, formal analysis, software, resources, data curation.

## Funding

The authors have nothing to report.

## Ethics Statement

Ethical approval for this study was obtained from the College of Nursing Ethical Committee at Hawler Medical University (Registration No. 2435, dated August 22, 2024), as well as from the General Directorate of Health, Erbil, and Hawler Teaching Hospital, in accordance with the Declaration of Helsinki.

## Conflicts of Interest

The authors declare no conflicts of interest.

## Transparency Statement

The lead author Sideeq Sadir Ali affirms that this manuscript is an honest, accurate, and transparent account of the study being reported; that no important aspects of the study have been omitted; and that any discrepancies from the study as planned and as registered have been explained in Section 2.13.

## Supporting information


Supporting File 1

Supporting File 2

Supporting File 3

Supporting File 4

## Data Availability

The data that support the findings of this study are available from the corresponding author upon reasonable request. The ethical approval document, the knowledge questionnaire and its scoring key, and the completed TREND and TIDieR checklists are provided as Supporting File.
