# Supplementary material for: A Standardized Perioperative Care Program and Postoperative Pain, Disability, and Knowledge Among Patients Undergoing Lumbar Spine Surgery in Erbil, Iraq: A Non‐Randomized Controlled Study
Source: Health Sci Rep. 2026 Sep 9;9(9):e73213. doi: 10.1002/hsr2.73213 (PMC13555270; doi:10.1002/hsr2.73213)
Supplement: Supplementary file 1 [file HSR2-9-e73213-s001.docx]

**Supplementary Files S4–S8. Additional tables, diagnostics, and protocol material**

**Supplementary File S4. Baseline characteristics of allocated and non-allocated participants**

Twenty-five participants who completed baseline assessment were not allocated because no matched counterpart was available on surgery type, age band, sex, and educational level. Their baseline characteristics are compared with those of the 70 analysed participants below.

| **Characteristic** | **Analysed (n = 70)** | **Not allocated (n = 25)** | **p** |
| --- | --- | --- | --- |
| Age, mean ± SD (years) | 47.76 ± 10.10 |  |  |
| Female, n (%) | 56 (80.0) |  |  |
| Illiterate, n (%) | 32 (45.7) |  |  |
| BMI, mean ± SD (kg/m²) | 30.74 ± 4.59 |  |  |
| Spinal fusion, n (%) | 50 (71.4) |  |  |
| Baseline pain, median (IQR) | 9 (8–10) |  |  |
| Baseline ODI %, median (IQR) | 74 (64–82) |  |  |
| Baseline knowledge, median (IQR) | 40 (37–45) |  |  |

The values for the analysed sample are pooled across the two study groups from Tables 1 and 2 of the main manuscript. The corresponding values for the 25 participants who completed baseline assessment but could not be matched to a counterpart are drawn from the same baseline dataset and are entered in the adjacent column, with group comparison by independent-samples t-test or chi-square test as appropriate.

**Supplementary File S5. Responder proportions stratified by baseline-severity tertile (exploratory)**

| **Outcome domain** | **Baseline-severity stratum** | **Int. n** | **Con. n** | **Int. responders n (%)** | **Con. responders n (%)** | **RD (95% CI)** |
| --- | --- | --- | --- | --- | --- | --- |
| Pain (≥2-point reduction) | UPAT ≤ 8 | 11 | 10 | 11 (100.0) | 5 (50.0) | 0.50 (0.20–0.80) |
|  | UPAT = 9 | 13 | 14 | 13 (100.0) | 10 (71.4) | 0.29 (0.06–0.51) |
|  | UPAT = 10 | 11 | 11 | 11 (100.0) | 8 (72.7) | 0.27 (0.01–0.54) |
| Disability (≥25.6 percentage-point reduction) | ODI ≤ 66% | 10 | 11 | 9 (90.0) | 4 (36.4) | 0.54 (0.18–0.89) |
|  | ODI 68–78% | 16 | 13 | 15 (93.8) | 5 (38.5) | 0.55 (0.27–0.83) |
|  | ODI ≥ 80% | 9 | 11 | 9 (100.0) | 6 (54.5) | 0.45 (0.16–0.75) |
| Knowledge (≥15-point gain) | ≤ 38 | 14 | 9 | 14 (100.0) | 1 (11.1) | 0.89 (0.66–1.00) |
|  | 39–43 | 13 | 13 | 13 (100.0) | 1 (7.7) | 0.92 (0.78–1.00) |
|  | ≥ 44 | 8 | 13 | 7 (87.5) | 2 (15.4) | 0.72 (0.40–1.00) |

*Note: RD = risk difference; CI = 95% confidence interval (Newcombe method). ODI strata are expressed on the 0–100% scale and correspond to the tertiles of the pooled preoperative distribution reported previously on the 0–50 summed scale. Stratum sizes of 8–16 participants per cell give very imprecise estimates; these results are descriptive and hypothesis-generating only, and relative risks and numbers needed to treat have been omitted for this reason.*

**Supplementary File S6. Regression model diagnostics**

| **Diagnostic** | **Value / result** |
| --- | --- |
| Observations included | 70 (complete cases) |
| Predictors in final model | 8 |
| Observations per predictor | ≈ 8.8:1 |
| Variance inflation factors | 1.08–1.42 (no multicollinearity) |
| Residual vs fitted plot | No systematic pattern; homoscedasticity assumption acceptable |
| Normal P–P plot of residuals | Points lie close to the diagonal; no substantive departure from normality |
| Linearity of predictor–outcome relationships | Confirmed by partial regression plots |
| Influential cases | No case exceeded a Cook's distance of 1 |
| Variable selection | None; all predictors pre-specified and forced into their blocks |

*Note: diagnostics were obtained in SPSS v27.0 (collinearity diagnostics, casewise diagnostics, standardized residual versus predicted value plots, normal probability plots, and partial regression plots). The model is reported as exploratory: with eight predictors and 70 observations the coefficients are likely to be optimistic and should not be used for individual prediction.*

**Supplementary File S7. Exploratory decomposition of the association between group, knowledge change, and outcome change (non-causal)**

The analysis below was presented in the previous version of the manuscript as a causal mediation analysis. It is retained here only as a descriptive decomposition of observed associations. The assumptions required for causal interpretation — no unmeasured confounding of the exposure–intermediate, exposure–outcome, and intermediate–outcome relationships — cannot be defended in a small non-randomized study in which the exposure, the intermediate variable, and the outcomes were assessed by the same team within the same care relationship. Proportions "mediated" have therefore been removed, and no causal inference is drawn.

| **Path** | **Estimate** | **SE** | **95% CI (bootstrap)** | **p** |
| --- | --- | --- | --- | --- |
| Model 1: group → knowledge change → pain change |  |  |  |  |
| Direct path (c′) | 2.18 | 0.34 | 1.51–2.85 | <0.01 |
| Indirect path (a×b) | 1.24 | 0.21 | 0.86–1.68 | <0.01 |
| Total (c) | 3.42 | 0.35 | 2.74–4.10 | <0.01 |
| Path a (group → knowledge) | 26.80 | 1.32 | 24.19–29.41 | <0.01 |
| Path b (knowledge → pain) | 0.05 | 0.01 | 0.03–0.06 | <0.01 |
| Model 2: group → knowledge change → disability change |  |  |  |  |
| Direct path (c′) | 12.47 | 1.52 | 9.45–15.49 | <0.01 |
| Indirect path (a×b) | 7.36 | 1.18 | 5.24–9.78 | <0.01 |
| Total (c) | 19.83 | 1.54 | 16.78–22.88 | <0.01 |
| Path a (group → knowledge) | 26.80 | 1.32 | 24.19–29.41 | <0.01 |
| Path b (knowledge → disability) | 0.28 | 0.04 | 0.19–0.36 | <0.01 |

*Note: SE = standard error; CI = confidence interval; bootstrapped structural equation models, 5,000 resamples. Model fit: χ²/df = 1.42 and 1.38; CFI = 0.98 and 0.99; TLI = 0.97 and 0.98; RMSEA = 0.08 and 0.07; SRMR = 0.04. Disability values are on the 0–50 summed scale as originally estimated; on the 0–100% scale the coefficients for disability are doubled. Adequate model fit does not establish causal identification.*

**Supplementary File S8. Ethical approval and statement on prespecification**

The ethical approval issued by the College of Nursing Ethical Committee at Hawler Medical University (Reg. No. 2435, 22 August 2024) is provided with this submission as a separate file. Approval was granted on the basis of the research proposal submitted to the committee before recruitment began.

No standalone protocol document was registered and no written statistical analysis plan was filed with the committee, because neither was required for non-randomized nursing evaluations at the institution at that time. The authors report this openly rather than describing analyses as prespecified when no written plan exists. The following statement distinguishes what was fixed in advance from what was decided after data collection:

1. Fixed before recruitment, in the approved research proposal: the study design; the eligibility criteria; the intervention and its schedule; the comparison condition; and the three outcomes — pain, functional disability, and patient knowledge — with measurement at baseline and at two months.

2. Not fixed in a written plan before data collection: all statistical analyses. The descriptive between-group and within-group comparisons follow directly from the outcomes and timing specified in the proposal; every other analysis was decided after the data were collected.

3. Added in response to peer review: the adjusted change-from-baseline analysis (analysis of covariance and linear mixed model) reported as the primary analysis in this revision. It is post-hoc.

4. Conducted after data collection and reported as exploratory throughout: the responder / minimal important difference analysis, the hierarchical regression, and the analysis of knowledge change as an intermediate variable. The clinical decision-support framework previously derived from these data has been removed from the manuscript.

5. No outcomes were collected beyond the three specified before recruitment, and no participant was excluded on the basis of an outcome value.

6. The study was registered with ClinicalTrials.gov (NCT07459985) on 04/03/2026, after completion of data collection.
