# Supplementary material for: A Standardized Perioperative Care Program and Postoperative Pain, Disability, and Knowledge Among Patients Undergoing Lumbar Spine Surgery in Erbil, Iraq: A Non‐Randomized Controlled Study
Source: Health Sci Rep. 2026 Sep 9;9(9):e73213. doi: 10.1002/hsr2.73213 (PMC13555270; doi:10.1002/hsr2.73213)
Supplement: Supplementary file 2 [file HSR2-9-e73213-s002.docx]

**Supplementary File S2. Intervention description (TIDieR checklist) and detailed description of usual care**

**Part A. TIDieR checklist**

| **Item** | **Description as implemented** |
| --- | --- |
| 1. Brief name | Standardized perioperative care programme for lumbar spine surgery. |
| 2. Why (rationale) | Structured perioperative preparation and reinforcement are hypothesised to improve self-management, adherence to activity restrictions, and realistic expectations, which are associated with postoperative pain and function. Structured perioperative education is not routinely provided in the study unit. |
| 3. What (materials) | Illustrated booklet in Kurdish and Arabic; instructional videos; standardized teaching plan; session-completion checklist. Content adapted from international postoperative spine-care guidance and reviewed by 24 clinical experts for accuracy and cultural relevance. |
| 4. What (procedures) | Preoperative: spinal anatomy, surgical goals, pain management, preparatory breathing and mobility exercises. Postoperative: pain management, wound care, recognition of infection, safe mobility and transfers, gradual activity progression, brace and compression-stocking use, nutrition, and return to work and daily activities. |
| 5. Who provided | One trained nurse researcher delivered all sessions throughout the study, using a fixed protocol. |
| 6. How (mode) | Face-to-face individual teaching, supported by printed and audiovisual material; family member present at follow-up sessions. |
| 7. Where | Neurosurgery Unit of Hawler Teaching Hospital (inpatient component) and the participant's home (home visits and follow-up sessions), Erbil, Iraq. |
| 8. When and how much | One preoperative session of 45–60 minutes; daily inpatient reinforcement until discharge; home visits every other day until suture removal (10–14 days); four structured follow-up sessions at 12–14 days and at 4, 6, and 8 weeks postoperatively, each 30–40 minutes. Estimated total contact 8–10 hours per participant. |
| 9. Tailoring | No tailoring: identical content and schedule for all intervention participants. |
| 10. Modifications | No modifications were made to the programme during the study period. |
| 11. How well (planned) | Fidelity monitored using a session-completion checklist recording delivery of each scheduled component, completed by the provider after every contact. |
| 12. How well (actual) | All 35 intervention participants received the preoperative session, daily inpatient reinforcement, the scheduled home visits until suture removal, and all four structured follow-up sessions. No participant discontinued the programme and none was lost to follow-up. |

**Part B. Detailed description of usual care (control condition)**

| **Element** | **Usual care as delivered in the study unit** |
| --- | --- |
| Preoperative contact | Verbal explanation of the planned procedure and its risks by the admitting surgeon at the time of consent, typically 5–10 minutes. No structured educational session, booklet, or video. |
| Inpatient education | Bedside verbal instruction on wound care, analgesia, and mobilisation given by ward nurses as part of routine nursing care, not standardized and not documented as an educational intervention. |
| Physiotherapy | Assistance with first mobilisation by a physiotherapist, as for all postoperative patients. |
| Discharge information | Written prescription and a standard discharge slip stating the date of suture removal and the date of routine outpatient review. No structured self-care content. |
| Follow-up | One outpatient visit for suture removal at 10–14 days and one further routine surgical review. No home visits, no scheduled telephone contact, no additional educational sessions. |
| Research contact | Baseline and two-month assessments only, containing no educational content. |
| Estimated total non-surgical professional contact | Approximately 15–30 minutes, compared with approximately 8–10 hours in the intervention group. |

The asymmetry in professional contact between the two conditions is reported in the manuscript as a central limitation: the study compares an intensive structured care pathway with low-intensity usual care, and attention and co-intervention effects cannot be separated from the effect of the educational content.
