# Supplementary material for: A Standardized Perioperative Care Program and Postoperative Pain, Disability, and Knowledge Among Patients Undergoing Lumbar Spine Surgery in Erbil, Iraq: A Non‐Randomized Controlled Study
Source: Health Sci Rep. 2026 Sep 9;9(9):e73213. doi: 10.1002/hsr2.73213 (PMC13555270; doi:10.1002/hsr2.73213)
Supplement: Supplementary file 3 [file HSR2-9-e73213-s003.docx]

**Supplementary File S1. Postoperative Spine Care Knowledge Questionnaire (36 items): instrument, scoring key, and development record**

**1. Purpose and development**

The questionnaire was developed for this study because no validated instrument assessing patient knowledge of postoperative lumbar spine self-care was available in Kurdish or Arabic. Items were generated from a structured review of the literature and of existing patient-education materials for lumbar spine surgery, and were mapped to nine content domains derived from the intervention curriculum.

Content validity was assessed by 25 experts (neurosurgeons, neurosurgical and orthopaedic nurses, physiotherapists, and nurse educators), who rated each item for relevance on a 4-point scale. Items with an item-level content validity index below 0.78 were revised or replaced. The final scale-level content validity index (average) was 0.91. The instrument was written in English, translated into Kurdish and Arabic and independently back-translated, and reconciled by the research team. Face validity and clarity were tested in a pilot of 18 patients (June 2–July 13, 2024), after which minor wording changes were made. Internal consistency was Cronbach's α = 0.833 in the pilot and 0.85 in the main sample.

**2. Structure and scoring**

| **Domain** | **Items** | **Item numbers** |
| --- | --- | --- |
| A. Spinal anatomy | 4 | 1–4 |
| B. Disc pathology | 4 | 5–8 |
| C. Incision and wound care | 4 | 9–12 |
| D. Pain management | 4 | 13–16 |
| E. Nutrition | 4 | 17–20 |
| F. Braces and compression stockings | 4 | 21–24 |
| G. Postoperative physical activity | 4 | 25–28 |
| H. Activities of daily living | 4 | 29–32 |
| I. Recommended exercises | 4 | 33–36 |

Each item has one correct option. A correct response scores 1 and an incorrect or "do not know" response scores 0, giving a raw score of 0–36. The raw score is linearly transformed to a 0–100 scale (raw × 100 / 36) for analysis and reporting. The questionnaire is interviewer-administered, takes approximately 15 minutes, and was completed at baseline and at two-month follow-up. Correct answers are marked with an asterisk (*) in the key below; the asterisks do not appear on the version administered to participants.

**3. Instrument and answer key**

***Domain A — Spinal anatomy***

**1. The lumbar spine is located in the:**

a) Neck

b) Upper back

c) Lower back *

d) Pelvis

**2. How many vertebrae form the lumbar spine?**

a) Three

b) Five *

c) Seven

d) Twelve

**3. The main function of the intervertebral disc is to:**

a) Produce blood cells

b) Act as a shock absorber between the vertebrae *

c) Store calcium

d) Connect the ribs to the spine

**4. The spinal cord and nerve roots are protected mainly by:**

a) The back muscles

b) The skin

c) The bony vertebral canal *

d) The intervertebral disc alone

***Domain B — Disc pathology***

**5. A herniated ("slipped") disc means that:**

a) A vertebra is broken

b) The inner material of the disc pushes out and may press on a nerve *

c) The spine is infected

d) A back muscle is torn

**6. Pain that travels from the lower back down the leg is called:**

a) Sciatica *

b) Migraine

c) Arthritis

d) Muscle cramp

**7. Which of the following most increases the risk of disc problems?**

a) Drinking water regularly

b) Repeated bending and lifting heavy objects *

c) Sleeping eight hours a night

d) Walking on level ground

**8. New numbness or weakness in the leg after surgery should be:**

a) Ignored, as it is always temporary

b) Treated with massage only

c) Reported promptly to the surgical team *

d) Treated with extra painkillers at home

***Domain C — Incision and wound care***

**9. The surgical wound should be kept:**

a) Moist at all times

b) Clean and dry *

c) Covered with oil

d) Uncovered and unwashed

**10. Which combination of signs suggests wound infection?**

a) Redness, swelling, warmth, and discharge *

b) Mild itching after three weeks

c) A thin dry scar

d) Slight bruising on the first day

**11. Sutures or staples are usually removed:**

a) Two days after surgery

b) Ten to fourteen days after surgery *

c) Two months after surgery

d) They are never removed

**12. Before touching the wound or changing the dressing you should:**

a) Wash your hands thoroughly *

b) Apply powder to the area

c) Rub the area to improve circulation

d) Remove the dressing quickly without preparation

***Domain D — Pain management***

**13. Pain medication after surgery should be taken:**

a) Only when the pain becomes unbearable

b) As prescribed, before the pain becomes severe *

c) At double dose at night

d) Whenever a family member advises

**14. Which is a non-drug method that helps control postoperative pain?**

a) Relaxation and controlled breathing *

b) Complete bed rest for one month

c) A hot bath directly on the wound

d) Smoking

**15. Applying a cold pack near (not on) the operated area may:**

a) Increase the risk of infection

b) Help reduce swelling and pain *

c) Always delay healing

d) Cause a fracture

**16. You should contact the surgical team if the pain:**

a) Improves gradually each week

b) Becomes suddenly severe or is accompanied by new weakness, fever, or wound discharge *

c) Appears briefly after walking

d) Decreases after taking the prescribed medicine

***Domain E — Nutrition***

**17. A diet rich in protein after surgery mainly helps to:**

a) Increase pain tolerance

b) Promote wound healing and tissue repair *

c) Reduce the need for sleep

d) Prevent sciatica

**18. To prevent constipation after surgery you should:**

a) Reduce fluid intake

b) Increase fibre and fluids and mobilise as advised *

c) Avoid all fruit and vegetables

d) Remain in bed as long as possible

**19. Which nutrients are particularly important for bone health?**

a) Calcium and vitamin D *

b) Carbonated soft drinks

c) Fried foods

d) Tea only

**20. Excess body weight after spine surgery:**

a) Has no effect on the spine

b) Increases mechanical load on the spine and may slow recovery *

c) Speeds up healing

d) Prevents postoperative pain

***Domain F — Braces and compression stockings***

**21. The main purpose of the back brace is to:**

a) Reduce body weight

b) Support the spine and limit harmful movement during healing *

c) Treat wound infection

d) Replace the need for exercise

**22. The brace should usually be applied:**

a) After standing up

b) While lying down, before getting out of bed *

c) Only at night

d) Only during outpatient visits

**23. Compression (anti-embolism) stockings are used mainly to:**

a) Reduce the risk of blood clots in the legs *

b) Reduce back pain

c) Keep the feet warm

d) Protect the surgical wound

**24. Compression stockings should be worn:**

a) Loosely, folded at the top

b) Smooth and wrinkle-free, for the period instructed *

c) Only while walking

d) Cut open for comfort

***Domain G — Postoperative physical activity***

**25. Getting out of bed early, as instructed by the team, helps to:**

a) Increase the risk of wound rupture

b) Prevent chest infection and blood clots *

c) Delay recovery

d) Cause a new disc herniation

**26. Which activity should be avoided in the early weeks after surgery?**

a) Walking on a flat surface

b) Bending forward, twisting, and lifting heavy objects *

c) Standing up slowly

d) Ankle exercises in bed

**27. The safest way to get out of bed after lumbar surgery is to:**

a) Sit straight up quickly from lying

b) Roll onto the side keeping the back straight, then push up with the arms *

c) Twist the trunk and swing the legs

d) Pull yourself up using the back muscles

**28. Walking distance after discharge should be:**

a) Increased gradually as tolerated *

b) Maximal from the first day

c) Avoided entirely for two months

d) Limited to climbing stairs

***Domain H — Activities of daily living***

**29. When picking up an object from the floor you should:**

a) Bend from the waist with straight legs

b) Bend the knees and keep the back straight *

c) Twist while lifting

d) Lift quickly with one hand

**30. Prolonged sitting in the early postoperative weeks should be:**

a) Unlimited

b) Limited, with regular position changes and lumbar support *

c) Done on a very soft sofa

d) Done on the floor

**31. Driving a car after lumbar surgery should be resumed:**

a) On the first day at home

b) Only after approval by the surgeon *

c) Never again

d) As soon as there is no pain at rest

**32. Which sleeping position is generally recommended?**

a) Face down with a high pillow

b) On the back with a pillow under the knees, or on the side with a pillow between the knees *

c) Sitting upright all night

d) On a very soft mattress

***Domain I — Recommended exercises***

**33. Ankle pumping exercises after surgery are performed mainly to:**

a) Strengthen the neck

b) Improve leg circulation and reduce the risk of clots *

c) Heal the wound

d) Improve vision

**34. Deep breathing and supported coughing exercises help to:**

a) Prevent chest complications after surgery *

b) Increase back pain

c) Loosen the sutures

d) Replace the need to walk

**35. Prescribed exercises should be:**

a) Continued to the point of severe pain

b) Performed regularly as instructed and stopped if severe pain occurs *

c) Performed once and then discontinued

d) Avoided until all pain has gone

**36. Which exercise is usually advised in the early postoperative period?**

a) Sit-ups

b) Heel slides and gentle walking *

c) Heavy weightlifting

d) Repeated trunk twisting
