# Supplementary material for: A Standardized Perioperative Care Program and Postoperative Pain, Disability, and Knowledge Among Patients Undergoing Lumbar Spine Surgery in Erbil, Iraq: A Non‐Randomized Controlled Study
Source: Health Sci Rep. 2026 Sep 9;9(9):e73213. doi: 10.1002/hsr2.73213 (PMC13555270; doi:10.1002/hsr2.73213)
Supplement: Supplementary file 4 [file HSR2-9-e73213-s004.docx]

**Supplementary File S3. TREND checklist for reporting non-randomized evaluations**

Des Jarlais DC, Lyles C, Crepaz N; TREND Group. Am J Public Health. 2004;94(3):361–366.

| **No.** | **TREND item** | **Reported in** |
| --- | --- | --- |
| 1 | Title and abstract: sample described; design described as non-randomized | Title; Abstract (Method) |
| 2 | Introduction: scientific background, rationale, theories used | Section 1 |
| 3 | Participants: eligibility criteria, recruitment method, recruitment setting, catchment area | Sections 3.2, 3.3 |
| 4 | Interventions: details of intervention and comparison condition, provider, delivery, exposure quantity and duration | Sections 3.6, 3.7; Supplementary S2 |
| 5 | Objectives: specific objectives and hypotheses | Sections 1, 2 |
| 6 | Outcomes: definitions, measurement methods, validation, whether assessors were blinded | Sections 3.4, 3.5 |
| 7 | Sample size: how determined; interim analyses and stopping rules (none used) | Section 3.9 |
| 8 | Assignment method: unit of assignment, method used, inclusion of methods to reduce bias | Section 3.3 |
| 9 | Blinding (masking): whether participants, providers, and assessors were blinded | Sections 3.5, 3.8 (participants and provider not blinded) |
| 10 | Unit of analysis: unit used, and methods to match unit of analysis to unit of assignment | Sections 3.11a–b (individual) |
| 11 | Statistical methods: primary comparison, additional analyses, missing data, software | Sections 3.11a–f |
| 12 | Participant flow: enrolment, assignment, exposure, follow-up, analysis; flow diagram | Section 4.1; Figure 1 |
| 13 | Recruitment: dates defining periods of recruitment and follow-up | Sections 3.2, 3.3 |
| 14 | Baseline data: baseline demographic and clinical characteristics; comparability of groups | Sections 4.2, 4.3; Tables 1, 2; Supplementary S4 |
| 15 | Baseline equivalence: data on comparability and methods used to control for differences | Sections 4.2–4.4, 3.11b |
| 16 | Numbers analysed: number included in each analysis and whether analysis was by assignment | Sections 4.1, 3.11f; Figure 1 |
| 17 | Outcomes and estimation: results for each outcome, effect size estimate and precision, null findings | Sections 4.4–4.8; Tables 3–7 |
| 18 | Ancillary analyses: subgroup and exploratory analyses, indicating pre-specified vs exploratory | Sections 3.11c–e, 4.7–4.9; Supplementary S5–S7 |
| 19 | Adverse events: important adverse events or unintended effects | Sections 3.14, 4.10 |
| 20 | Interpretation: interpretation accounting for hypotheses, bias, imprecision, and multiplicity | Section 5 |
| 21 | Generalizability: applicability of findings, given the study population and setting | Section 5 |
| 22 | Overall evidence: general interpretation in the context of current evidence and theory | Sections 5, 6 |
